# Comparison of Right Anterolateral Thorocotomy with Standard Median Steronotomy for Mitral Valve Replacement

**Published:** 2013-03-15

**Authors:** Zamir Ahmad Shah, Abdual Gani Ahangar, Farooq Ahmad Ganie, Mohd Lateef Wani, Hafeezulla Lone, Nasir­ud­din Wani, Shadab Nabi Wani, Irteka Muzamil, Masaratul Gani

**Affiliations:** 1 Department of cardiovascular and thoracic surgery, SKIMS, Soura, Kashmir , India; 2 Department of Anesthesia, SKIMS, Soura, Kashmir, India; 3 J and K Health services,. SKIMS, Soura, Kashmir, India

**Keywords:** Thoracotomy, Median Sternotomy, Mitral Valve

## Abstract

**Obejectives:**

The objectives of this study were to compare and analyze the results of right anterolateral thoracotomy and median sternotomy approach for primary mitral valve replacement with reference to the exposure during Valve Replacement , length of surgical incision, mean cross clamp time, mean bypass time, intensive care unit (ICU) stay, hospitalization, overall comorbidity with sternotomy; sepsis, dehiscence, healing cosmetic issues and cost effectiveness.

**Methods:**

The present study comprised 68 patients with rheumatic mitral valve disease who underwent mitral valve replacement in the Department ofCardiovascular and Thoracic Surgery at Sher‑i‑Kashmir Institute of Medical Sciences from September 2009 to August 2011.

**Results:**

This study comprised 64 patients with 23 (35.9%) males and 41 (64.1%) females. Sternotomy group had 10 males (31.3%) and 22 females (68.7%). Thoracotomy group had 13 males (40.6%) and 19(59.4%) females. The length of incision between the two groups was statistically significant (P<0.0001). Mean incision length were 24.6±2.1 cm and 14.8±2.3 cm in sternotomy and thoracotomy respectively. Statistically significant difference regarding duration of ICU stay was found between the two groups (P<0.0001). Scar visibility was 100% in sternotomy and around 25% in thoracotomy( P<0.0001).

**Conclusions:**

Thoracotomy through a right anterolateral aspect was easy to perform while maintaining maximum security for the patients. Besides its satisfactory cosmetic result especially in female patients, this approach proved to have several advantages. It offers a better exposure to the mitral apparatus even in patients with small left, allowing easy mitral valve replacement which is apparent from the lower cross‑clamp time in the test group. The invaluable advantage of the above- mentioned thoracotomy is total eradication of the risk of deep sternal infection. The shorter hospital stay and cost effectiveness of thoracotomy approach are additional relief to the family.

## 1. Background

Heart valves function is to maintain pressure gradients between cardiac chambers and ensuring unidirectional flow of blood without reflux through the heart. Among the heart valves, the aortic and mitral valve, are by far most vulnerable to disease. In regard to public health, medical diagnostic and surgical technique mitral valve diseases have been considered as the most interesting human maladies to be treated in this century (1, 2). Rheumatic fever and rheumatic heart disease affect mitral valve by causing stenosis of both anteromedial and posterior commusures of the valve with subsequent mitral regurgitation. The compensatory mechanisms of ventricles permit the heart to tolerate these lesions for varying periods of time. Heart failure from mitral stenosis was well recognized by 19 th century and surgical correction began well before the heart lung machine was available (1,2). Valvular heart diseases may be considered as surgical illnesses. Lillehei repaired multiple valvular lesions through a right thoracotomy using cardiopulmonary bypass in 1956. A few years later, in 1961, A. Starr successfully replaced a mitral valve with a prosthetic valve (1,3).

In 1965 Jai­ Si Haung performed first open heart mitral valve replacement at Shanghai hospital, China. The ensuing years witnessed the rapid development of various valvular prostheses placed via a conventional approach,a full sternotomy with cardiopulmonary bypass (CBP) (1). However, over the past few decades, minimally invasive mitral valve surgery has grown in popularity and evolved significantly over the past 10 years and currently comprises a safe and efficient operation for most patients. At the Brigham and Women's Hospital (BWH), they did not involve a complete sternotomy but instead uses a partial sternotomy or limited thoracotomy incision (4). A variety of techniques have been described to reduce surgical approach in mitral valve surgery. The most common minimally­ invasive approach to the mitral valve includes a partial sternotomy and a right mini­thoracotomy. Either approach may be performed using standard conventional access, port access, indirect endoscopic methods and more recently robotic techniques (5). Although there has been great enthusiasm in recent years to perform mitral valve surgery through small multiple incisions using port access technique, however the procedure is costly, involves a relatively long training curve and leaves the patient with multiple scars in the chest and groin. Median sternotomy, which is generally used as a standard access for mitral valve operations has a significant risk of postoperative instability/osteomyelitis of the sternum. Moreover, the resulting large scar is associated poor cosmetic outcome and especially in young women may have adverse psychological consequences.Sternotomy independently increases the risk of postoperative morbidity and mortality in patients of cardiac surgery especially when associated with other comorbid conditions like diabetes (6). Approach to mitral valve via right anterolateral thoracotomy is not new. The principle of this kind of Approach is to reduce the morbidity and the cost, speed hospital discharge and shorten the rehabilitation time (7, 8). Right anterolateral thoracotomy has been recommended as an alternative approach to standard median sternotomy for patients undergoing mitral valve replacement.The purpose of this study was to compare right anterolateral thoracotomy with standard median sternotomy for mitral valve replacement in terms of cost benefits and other variables including cosmetic aspects.

## 2.Materials and Methods

The study was carriedout in the Department of Cardiovascular and Thoracic Surgery Sher­i­ Kashmir Institute of Medical Sciences and comprised of a prospective study from September 2009 to August 2011. The patients were followed for a period of 2­2.5years.

In the prospective study, patients were examined and evaluated A detailed clinical examination was carried out with special references to cardiovascular system .Patients were randomly allocated into two groups using computer generated random numbers. who required mitral valve replacement (MVR) according to the ACC/AHA guidelines (9) were included in the study. The groups were matched with respect to age, sex, NYHA Class, and ejection fraction. Length of incision, surgical exposure, mean crossclamp time, mean bypass time, ICU stay, hospital stay, overall comorbidity with sternotomy. Sepsis, dehiscence, healing and cosmetic quality were studied for comparison. Their follow up information was obtained prospectively by observing patients in follow up clinic. The study comprised 64 patients with mitral valve disease undergoing MVR.

*2.1 Surgical procedure*

The study group comprised 50% of patients who underwent MVR via right anterolateral thoracotomy and contol group including another 50% were subjected to MVR via median sternotomy. The same general anesthetic techniques with routine arterial and venous monitoring were utilized for both groups.

In regard to thoracotomy group incision was made in the right sub-mammary fold starting 3–5 cm from the lateral border of the sternum. The breast tissue in females was gently mobilized and the right chest cavity was entered through the fourth intercostal space. Aortic and bicaval cannulation was then performed in the usual manner and cardiopulmonary bypass instituted. After cooling to 32°C, the aorta was cross clamped using a long curved aortic clamp in order to keep it out of the surgeon’s field, and aortic root blood cardioplegia was delivered. The left atrium was opened through an incision posterior and parallel to the interatrial groove that accessed the mitral valve. The diseased mitral valve was excised and then replaced by a prosthetic valve secured to annulus using continuous 20 prolene suture. The left atriotomy was closed by a single layer of 3/0 silk suture and de­airing was performed through the suture line before removing the aortic cross­clamp. Following re-warming to 37°C, the heart was allowed to take over the circulation. Decannulation was then performed and the suture line secured before giving the protamine. This was followed by complete closure of the pericardium by continuous sutures, leaving a small drain. The chest was then closed in layers leaving a separate thoracic drain.

As for the control group, the approach was through the standard median sternotomy, but otherwise the operative technique was essentially the same.

*2.2 Postoperative management*

Patients were electively ventilated overnight. Post extubation patients were shifted from ICU after completely assessing the general condition and hemodynamics of the patients along with baseline investigations and blood gases. Oral anticoagulant was started on second postoperative day with acenocoumarol to maintain a n International normalized ratio(INR) of 2.02.5.

Intravenous antibiotics, a combination of ceftriaxone/sulbactam and amikacin were administered during hospitalization and changed according to clinical situation. Intravenous antibiotics were continued during the hospital stay.

*2.3 Statistical Analysis*

The results were presented as mean ±2 standard deviation. Statistical analysis was performed between the two groups using the independent sample t­test and Chi square test. A significant difference was recorded if the P value was less than or equal to 0.05. SPSS ­17 for Windows was used for statistical analysis.

## 3. Results

All patients patients were operated for mitral valve disorder and underwent mitral valve replacement. One patient from thoracotomy group and 3 patients from sternotomy group expired during the course of study and were excluded from the study. Study was completed in 64 patients.

Mean age in sterntomy group was 42.56 ± 6.2 years and in thoracotomy group it was 44.41 ± 8.2 years. In our study 64 patients 23 (35.9%) were males and 41 were females (64.1%). Sternotomy group had 10 males (31.3%) and 22 females (68.7%). Thoracotomy group had 13 males (40.6%) and 19 females (59.4%).

In our study 20 (62.5%) and 24 (75%) of the cases were class III in sternotomy and thoracotomy groups respectively while the remaining patients were class IV (Table 1).

**Table 1.** NYHA Class of 64 Patients

Group	NYHA Class		Total
	III	IV	
Sternotomy	20 (62.5%)	12 (37.5%)	32 (100.0%)
Thoracotomy	24 (75.0%)	8 (25.0%)	32 (100.0%)
Total	44 (68.7%)	20 (31.3%)	64 (100.0%)

The two groups were comparable with respect to ejection fraction. Most patients had an ejection fraction between 30%­-50%; 68.7% in sternotomy and 78.1% in thoracototmy groups.

A statistically significant difference in length of incision was found between the two groups (*P*<0.001). Mean incision length were 24.6±2.1 cm and 14.8±2.3 cm in sternotomy and thoracotomy respectively. A statistically significant difference in cross clamp time was found between the two groups (P=0.047). Cross clamp time was 45.3±8.3 minutes in sternotomy group and 41.7±5.7 minutes in thoracotomy group.

Total bypass time for sternotomy was 82.3±9.1 and for thoracotomy 83.3±10.7 hours(P=0.69). Total operating time was 4.6±0.3 and 4.7±0.4 hours for sternotomy and thoracotomy respectively (P=0.245).

Statistically significant difference in duration of ICU stay was seen between the two groups.

Duration of ICU stay was 21.9±3.7 hours in sternotomy and 17.1±4.2 hours in thoracotomy groups (P <0.0001).

Difference in Duration in post operative hospital stay was statistically significant (P<0.001) with 11.1± 1.8 days and 9.2±1.1 days for sternotomy and thoracotomy respectively.

Wound infection was seen in 3 (9.3%) patients and in 1(3.1%) cases of sternotomy and thoracotomy groups respectively. Wound dehiscence was found in one patient of sternotomy group. Scar visibility was 100% in sternotomy and around 25% in thoracotomy groups (*P *<0.0001).

 Scar complication was found in18 (56.3%) patients of sternotomy and in only 2 (6.3%) of thoracotomy groups. Scar hypertrophy and stretching was seen more frequently in sternotomy group (31.3% and 21.8% respectively). Thorocotomy had an only incidence of 3.1% for each of these complications. 80%of females in thorocotomy group were satisfied with their scar appearance and cosmetic quality.

Postoperative drug cost in sternotomy group was 10.9±2.4 and in thoracotomy group 9.2±1.5 thousand( Indian Rupies) (*P*=0.002). There was no Conversion during surgery from thorocotomy to sternotomy for need of adequate exposure at the time of valve replacement.

## 4. Discussion

This study was conducted in the Department of Cardiovascular and Thoracic Surgery, Sher-i- Kashmir Institute of Medical Sciences, Soura. The patients in study were matched with respect to age, sex, NYHA class and ejection fraction and randomized in two groups using computer generated numbers. Median sternotomy, which generally is used as a standard access to mitral valve operations, carries a significant risk of postoperative infection and dehiscence. Moreover, especially in young women, the resulting large scar is of a poor cosmetic quality that may have adverse psychological consequences (5). These difficulties may be avoided by the use of a less invasive approach consisting of a limited anterolateral thoracotomy with standard cannulation. We studied whether such complications can be addressed by using right anterolateral thoracotomy, with simultaneous comparison of the procedure with certain intraoperative and post operative parameters. The patients in two groups were similar with respect to mean age, which was 42.5±6.2 years in sternotomy and 44.4±8.2 years in thoracotomy group. Patients may remain asymptomatic for many years as long as mitral stenosis (MS) is mild and not accompanied by more than mild mitral regurgitation(MS). Moreover, in developing countries, rheumatic MS manifests 10-30 years after the initial rheumatic insult to the mitral valve. Similar results were reported by Jonathan R. Carapetis (10). Karen Sliwa et al reported the highest prevalence of rheumatic heart disease in females at 45 to 54 years of age and in males aged from 55 to 64 years (2). In our study, most patients were females, and 68.7% and 59.4% were in sternotomy and thoracotomy groups respectively. Rheumatic fever affects both men and women equally, but MS and mitral regurgitation (MR) is more common among females with rheumatic fever. This was consistent with the study performed by A S Kumar et al. (11) at AIIMS on 38 patients (34 females and 4 males) who underwent mitral valve surgery through a limited right anterior thoracotomy. Srivastava AK (2,12) studied 52 patients among them 30 were females and 22 were males. In another study performed by Yugal K. Mishra et al. two-thirds of patients who came for mitral valve surgery were young women (12).

In our study all patients belonged to NYHA class III and IV which is consistent with that of Thompson et al. who found that 86% of patients belonged to NYHA class III and IV preoperatively. Twenty (63%) patients in sternotomy group were NYHA class III, and the remaining 37% were in class IV and 24 (75%) patients in thoracotomy group were class III while the rest were class IV. This is consistent with the study by Apostolos D, et al. (3) who reported that most of the patients referred for mitral valve surgery were in NYHA symptom class III and IV,although there were no statistically significant difference between the two groups.

Majority of the patients had an ejection fraction (EF) below 60% which is the indication for surgical intervention according to ACC/ AHA guidelines for management of patients with valvular heart disease. Sternotomy group had 7(21.9%) patients with EF >50%, 22 (68.7%) subjects with EF 30-50% and 3 (9.4%) having EF below 30%. In regard to thorocotomy group, EF were >50%, 30-50% and below30% in 5(15.1%),25 (78.1%) and2 (6.3%) patients respectively. Hence the groups were almost comparable with respect to ejection fraction.

Mean aortic crossclamp time was 45.3±8.3 minutes in sternotomy group and 41.7±5.7 minutes in thoracotomy group( p=.0.04). The observed values were well below the highest cutoff value for crossclamp time of 150 min which is significantly associated with postoperative morbidity, and particularly with postoperative stroke(13). The lesser cross clamp time in thoracotomy was due to easy accessibility to left atrium even with smaller atrial size. The observed crossclamp time was consistent with those t of Mohamed M. El-Fiky et al, studies (27±8 min) (14), Zapolanski A, et al. (70.0 min) (15), Riess FC, et al. (51.8 ± 21.9 min) (5), Grossi EA, et al. (92.0 hours) (16), Chiu KM et al. (43.7 min) (17), and Seeburger J, et al. (70±38min)(18) and virtuallycomparable with sternotomy group.

Mean bypass time in case of sternotomy was 82.3±9.1 hours and 83.3±10.7 in case of thoracotomy (P=0.69). The observed values were well below the highest cutoff value for total bypass time of 240 min and were significantly associated with postoperative morbidity, and in particular with postoperative stroke(13). The total bypass time in our study is comparable with those of Mohammed El-Fiky et al. (59±11 min) (14), Zapolanski A, et al. (77.0±25.8 hours)(19), Grossi EA, et al. (127.0 hours)(16), Aybek T,et al. (142.0±40 hours) (20), Chiu KM, et al. (91.1 hours)(17), and Seeburger J, et al. (121±38 hours) (18).

Total operating times were 4.6±.4 hours (276±24 min) and 4.7±0.4 hours (282±24 min) in sternotomy and thoracotomy groups respectively (P=0.245). William L. Holman et al. reported a mean operating time of 185±73 minutes (21). Riess FC et al reported an operating time of 211.9±36.0 minutes (5), which was not consistent with our experience. The difference was due to the total operating time starting with intubation of the patient , putting in central venous catheter and arterial line, positioning the patient until transferring the patient to ICU. However, in our study the two groups were comparable.

Overall surgical exposure was comparable between the two groups including cannulation of the vessels, aortic clamping, instituting cardioplegia using standard techniques as well as atriotomy, valve excision and replacement. There was no technical difficulty during aortic and major vessel cannulation in thoracotomy group. Left atrium was sufficiently exposed while the accessibility to valve during excision of valve and valve replacement was adequate. This was supported by conversion rate to sternotomy, for need of adequate exposure to valve apparatus was zero, and bycomparable results of bypass time, crossclamp time and total operating time between the two study groups. This observation is consistent with the studies by Kumar AS et al(11), Calleja F (7,22), Srivastava AK et al. (12), William L. Holman et al. (21), Yung MC et al. (22), Thompson MJ (23), Mohamed Abrahim Sewielam et al(24), and René Prêtre et al(25).

Statistically significant difference was seen in the duration of ICU stay (P<0.001).

In our study we had a choice of shifting the patients early to high dependency area of our general post operative ward. Duration ICU stay was 21.9±3.7 hours in sternotomy and 17.1±4.2 hours in thoracotomy. This is consistent with studies by Yung MC et al. (36.3±5.0 hours) (22), Reiss FC et al (28.8±9 hours) (5), Grossi EA, et al (hours) (16), Aybek T et al (18.0 hours) (20). Thoracotomy proved to be superior to sternotomy in terms of postoperative ICU stay.

Significant difference in duration of postoperative hospital stay was observed (P= 0.02) between the two groups (11.1±0.8 days and 9.2±1.7 days with sternotomy and thoracotomy respectively). Result was consistent with studies by Mohamed El-Fiky et al. (7±2) (14), Yung MC et al. (11.7±0.6 days) (22), Zopalinski et al. (8.1 days) (19), Riess FC et al. (7.8±2.2 days) (5), Grossi EA et al. (6.0 days)(16), Thompson MJ, et al. (12.0 days)(23),and Alexander Iribane (7.7±0.4 days) (26). Early ambulation, with subsequent early appreciation of patient's well-being and faster recovery reduced the overall hospital stay in thoracotomy group (15).

Thoracotomy approach utilizes a smaller incision length that improves the cosmetic result due to small scar which is less visible especially in females. The length of incision was significantly lesser in thoracotomy with a mean length of 15 cm as compared to 25cm for sternotomy (P<0.0001). El- Fiky et al. (14) reported an incision length of 12–15 cm in test group. Reduction in the size of the operative incision for cardiac valve surgery has been associated with reduced postoperative discomfort, shorter intensive care and hospital stay, earlier recovery and return to work, and an overall improvement in patient's satisfaction was reported by Apostolos D, et al. (3).

In our study 3 (9.3%) out of 32 patients in sternotomy group and 1 out of 32 (3.1%) cases in thoracotomy group had wound infection. Thoracotomy wounds were less prone to infection while sternal wounds were more vulnerable to infection. This was in agreement with the studies by Zapolanski A, et al. (19) and Aybek T, et al. (20) where wound dehiscence was seen in 1 patient of sternotomy and none in thoracotomy group. Antibiotics were routinely used in postoperative period, which was consistent with the study by Mohamed El-Fiky et al. (14).

Cosmetically the incision in thoracotomy group was better than the sternotomy group. The scar was 100% visible in frontal view in sternotomy while in thoracotomy it was less obvious and more laterally placed even in males. In females most of the incision length was hidden under the breast. This made it more convenient psychologically. According to EL-FIKY, et al., the wound was totally inapparent in their patients and more patients were in favor of this approach (14). The scar appearance was better in study group than in control group. Hypertrophy was observed in approximately 31.3% of sternotomy and 3.1% of thoracotomy groups. Other problems associated with sternotomy like keloid formation did not occur in thoracotomy group. Keloid formation occurred in a single case of sternotomy group (3.1%). Scar stretching occurred in 7 (21.8%) patiens of sternotomy and in one case of thoracotomy (3.1%). The cosmetic end product of the right thoracotomy technique was excellent, especially in young females. These features were in accordance with those of most studies (5,11,12, 14,19,20,22, 27-29).

Improved cosmetic quality is an undisputed advantage of minimally invasive valve surgery. Statistically no significant difference was found in the patient satisfaction when the two overall group populations were compared in regard to cosmetic aspects. However 15(80%) out of 19 females in thoracotomy group especially young women were satisfied with the postoperative scar appearance, whereas this was seen in only 12 out of 22 (54%) females in sternotomy group. On the other hand, there was no such difference found between males patients, with approximately 70% in each group. Higher percentage of patients in thoracotomy group appeared to be satisfied with the approach, and the rest remained indifferent to the type of approach used. This is consistent with the study by Cheng, Davy C. H. et al(30). In a study of patients having a right thoracotomy, Casselman et al.(31) reported that approximately 99% of patients with thoracotomy for MVR considered scar as aesthetically pleasing.

Postoperative drug cost was significantly lower in thoracotomy group than in sternotomy patients (P= 0.002). Less postoperative pain due to the use of intercostal nerve block reduced the need for frequent analgesia. Lesser inhibition of respiratory movements due to less pain helped early ambulation and rehabilitation, with subsequent lower cost of drug use and hospital stay. Post operative drug cost was about 15% less in the study group than in control group, a finding consistent with the study done by Melih Hulusi Us et al. (32) Chitwood et al. (33), Cohn et al. (34) and Navia and Cosgrove (35) who equated this to 34%, 20%, and 7% cost saving respectively. The reduced hospitalization associated with decreased hospital charge was not included in the study (9).
